# Reduction in new-onset diabetes mellitus after renal transplant with erythropoietin-stimulating agents: a retrospective cohort study

**DOI:** 10.1186/s40697-016-0114-9

**Published:** 2016-04-26

**Authors:** Tess Montada-Atin, Diana Choi, Minna Woo, Ravi Retnakaran, Michael Huang, G. V. Ramesh Prasad, Jeffrey S. Zaltzman

**Affiliations:** Renal Transplant Program, St. Michael’s Hospital, 61 Queen Street East, 9th Floor, Toronto, ON Canada M5C 2T2; Faculty of Medicine, University of Toronto, Toronto, Canada; Endocrinology and Metabolism, Toronto General Hospital, University Health Network, 200 Elizabeth Street, Toronto, ON Canada M5G 2C4; Leadership Sinai Centre for Diabetes, Mount Sinai Hospital, 60 Murray Street, Toronto, ON Canada M5T 3L9; Renal Transplant Research, St. Michael’s Hospital, 30 Bond Street, Toronto, ON Canada M5B 1W8; Lawrence S. Bloomberg Faculty of Nursing, University of Toronto, Toronto, Canada; Division of Endocrinology, University of Toronto, Toronto, Canada; Department of Medicine and Biophysics, University of Toronto, Toronto, Canada; Department of Medicine, University of Toronto, Toronto, Canada; Lunenfeld-Tanenbaum Research Institute, Mount Sinai Hospital, Toronto, Canada; Division of Nephrology, University of Toronto, Toronto, Canada

**Keywords:** NODAT, Diabetes, Renal transplant, Erythropoietin

## Abstract

**Background:**

Studies have shown that erythropoietin-stimulating agents (ESAs) protect mice against the development of diabetes through direct effects on pancreatic ß cells. However, the effect of ESAs on the incidence of diabetes in humans has not been well studied. It is unknown whether exposure to ESAs is associated with a reduced incidence of new-onset diabetes after transplant (NODAT).

**Objective:**

The objective of this study is to examine the relationship between ESA exposure post-renal transplant and the development of NODAT.

**Design:**

We performed a single center, retrospective cohort analysis.

**Patients:**

We compared patients who received a first live or deceased donor renal allograft, with any exposure to an ESA vs. those without such exposure and who developed NODAT and who did not. Patients with a prior history of diabetes mellitus or multi-organ transplant, including a second renal transplant were excluded.

**Measurements and methods:**

NODAT was defined based on the 2008 Canadian Diabetes Association criteria. Multivariate logistic regression analysis was performed to determine factors independently associated with NODAT.

**Results:**

One hundred thirty-two (29 %) patients were exposed to an ESA, four of which developed NODAT compared to 128 who did not develop NODAT (*p* < 0.0001). Of those not exposed to an ESA, 15 % (48/319) developed NODAT. By Fisher’s exact test, exposure to an ESA at any time post-transplant reduced the risk of developing NODAT; odds ratio (OR) = 0.08, 95 % confidence interval (CI) (0.018–0.352), *p* = 0.0008. Older age; OR = 1.41, 95 % CI (1.036–1.933), *p* < 0.02, higher random blood sugar at discharge; OR = 1.30, 95 % CI (1.077–1.57), *p* < 0.006 and deceased donor; OR 2.18 CI (1.009–4.729), *p* = 0.04 were associated with an increased risk of NODAT.

**Limitations:**

The limitations of this study include its retrospective nature, single center, and homogenous population; thus, generalizability of the results must be approached with caution.

**Conclusion:**

ESA exposure may be associated with a reduced incidence of NODAT in the post-renal transplant population. The role of ESA in preventing NODAT requires further investigation.

## Abrégé

**Mise en contexte:**

Des études ont démontré que les agents stimulant l’érythropoïèse (ASE) protègent les souris contre le développement du diabète par leur action directe sur les cellules bêta du pancréas. Toutefois, l’effet des ASE sur l’incidence du diabète chez l’humain n’a pas fait l’objet d’études approfondies. On ignore toujours si l’exposition aux ASE est associée à une réduction de l’incidence de l’apparition du diabète sucré post-transplantation (DSPT).

**Objectifs de l’étude:**

Cette étude visait à évaluer la relation entre l’exposition aux ASE à la suite d’une greffe de rein et la survenue du DSPT.

**Modèle d’étude:**

Il s’agit d’une étude de cohorte rétrospective qui s’est tenue dans un seul centre.

**Participants:**

Nous avons comparé les cas de patients qui recevaient une première allogreffe de rein, d’un donneur vivant ou décédé. Les comparaisons ont porté sur la présence ou l’absence d’une exposition aux ASE à la suite de la transplantation et sur le fait de développer ou non un DSPT conséquemment. Les patients ayant un historique de diabète sucré ou ayant subi une transplantation multiorganes dans le passé, y compris une deuxième greffe de rein, ont été exclus de l’étude.

**Méthodologie:**

La manifestation d’un DSPT a été déterminée selon des critères établis par l’Association Canadienne du Diabète en 2008. L’analyse par régression logistique multivariée a été utilisée pour déterminer les facteurs indépendamment liés au DSPT.

**Résultats:**

Sur les 132 patients (29 %) exposés aux ASE à la suite de la greffe, seuls quatre ont développé un DSPT (p < 0,000 1). Des patients qui n’avaient pas été exposés aux ASE, 15 % (48/319) ont développé un DSPT. Selon le test exact de Fisher, l’exposition à un ASE, peu importe le moment après la transplantation, a eu pour effet de réduire le risque de développer un DSPT [rapport de cote (RC) = 0,08 ; intervalle de confiance (IC) à 95 % (0,018-0,352), p = 0, 000 8]. Parmi les facteurs associés à un risque élevé de développer un DSPT, on note l’âge avancé du patient [RC = 1,41 ; IC à 95 % (1,036-1,933), p < 0,02], une glycémie aléatoire plus élevée à la sortie de l’hôpital [RC = 1,30 ; IC à 95 % (1,077-1,57), p < 0,006] et la transplantation d’un greffon provenant d’un donneur cadavérique [RC = 2,18 ; IC à 95 % (1,009-4,729), p = 0.04].

**Limites de l’étude:**

Cette étude est limitée par sa nature rétrospective et par le fait qu’elle ait été menée à l’intérieur d’un seul centre et au sein d’une population homogène. Par conséquent, la généralisabilité des résultats doit être abordée avec prudence.

**Conclusions:**

L’exposition aux ASE pourrait être associée à une réduction de l’incidence du DSPT au sein de la population des receveurs d’une greffe de rein. Le rôle des ASE dans la prévention du DSPT exige des recherches plus poussées.

## What was known before

We know in animal studies that erythropoietin-stimulating agents (ESAs) protect against the development of diabetes through direct effects on pancreatic ß cells and have been associated with improvement in glucose tolerance. Furthermore, patients with end-stage renal disease on hemodialysis treated with ESAs have been shown to have improvement in insulin sensitivity. However, the effect of ESA on the risk of diabetes in post-transplant patients has not been well studied.

## What this adds

This study is relevant as ESA and may be a novel therapy in the prevention of diabetes in the post-renal transplant population.

## Introduction

New-onset diabetes mellitus after renal transplantation (NODAT), a common post-transplant event, adversely affects graft and patient survival and quality of life [1, 2]. Evidence suggests that transplant recipients who develop NODAT are at greater risk of graft loss, rejection, and infection as well as diabetes-associated microvascular and macrovascular complications [3]. About one-third of nondiabetic renal transplant recipients develop persistently impaired glucose metabolism by 6 months post-transplantation [4–6]. However, the prevalence of NODAT at our institution is approximately 11.5 %. Risk factors such as advanced age, obesity, male gender, nonwhite ethnicity, a family history of diabetes, and the prescription of corticosteroids and calcineurin-inhibitors are well described [7, 8].

The prevalence of anemia post-transplant is dependent on the time of observation after transplantation [9, 10]. Although highly prevalent in the pre-transplant population with chronic kidney disease (CKD), anemia is a common event following renal transplantation as well. Studies report variable prevalence of 20 to 53 % at 1-year post transplant [11, 12]. Risk factors for post-transplant anemia include iron deficiency, graft rejection or dysfunction, erythropoietin (EPO) deficiency, viral infection, immunosuppression, infection prophylaxis medications, use of angiotensin converting enzyme inhibitors/angiotensin receptor blockers, low serum albumin, and older kidney donors [11, 12]. However, anemia in the immediate post-transplant period is almost always due to lower than normal hemoglobin targets in CKD and end-stage renal disease population on an ESA, surgical blood loss, and frequent phlebotomy [11]. Approximately, 10–15 % of post-renal transplant patients require an erythropoietin-stimulating agent (ESA) for the treatment of post-transplant anemia.

Erythropoietin (EPO), a hematopoietic growth factor, is largely produced in the kidney and has several biological functions, including the regulation of glucose metabolism [13]. Recombinant EPO forms or ESAs have been used to treat anemia in chronic kidney disease and other conditions [13, 14]. Patients with end-stage renal disease on hemodialysis treated with recombinant EPO have been shown to have improvement in insulin sensitivity [14–17]. ESA has been associated with improvement in glucose tolerance, both at the peripheral and skeletal tissue in mice models [13, 16, 18]. Furthermore, inhibition of gluconeogenesis and inflammation in the liver were observed with ESA in high-fat diet-fed mice [13]. Choi et al. and others demonstrated that EPO has a direct effect on pancreatic beta cells by increasing β-cell mass through anti-apoptotic, proliferative, and angiogenic mechanisms in mice models with type 1 and type 2 diabetes [14, 15, 19]. In Choi’s study, EPO was given three times weekly for 4 weeks compared to a saline control group. In both mice models, EPO lowered fasting blood glucose and slowed the loss of β-cell mass. In addition, by binding to erythropoietin receptors found in pancreatic cells, EPO significantly inhibited apoptosis induced by cytokines in vitro [20]. These studies suggest that EPO may reduce or prevent diabetes as it has protective effects on the beta cells as well as reducing insulin resistance.

A recent study by Shah et al., found no association with endogenous erythropoietin and glucose homeostasis in post-partum women with varying degrees of glucose intolerance [21]. However, the effect of exogenous ESA on the risk of diabetes in humans has not been well studied. It is unknown whether exposure to ESAs is associated with a reduced incidence of NODAT, or if ESAs play a role in preventing the development of NODAT. We therefore sought to examine the relationship between ESA exposure post-renal transplant and the development of NODAT.

## Methods

**Fig. 1:**
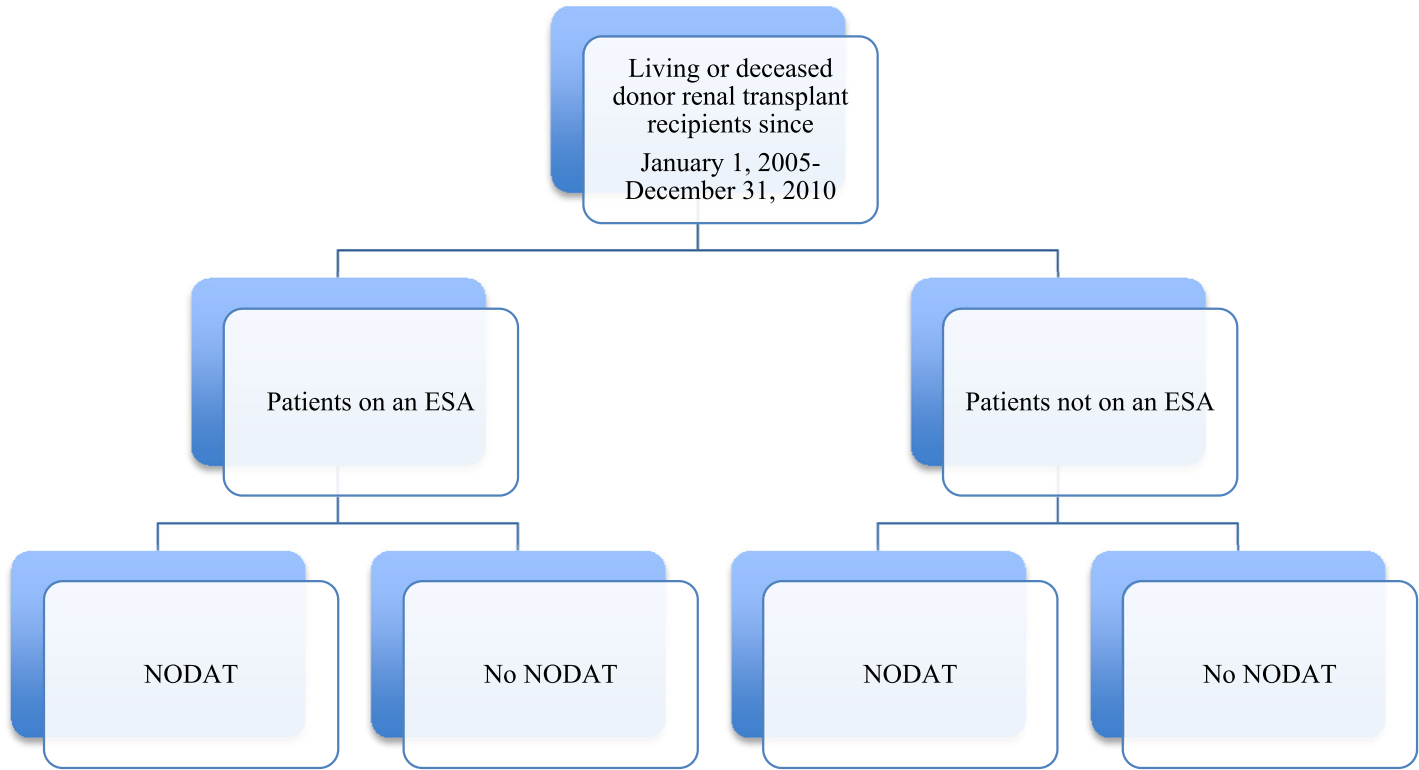
Study design

St. Michael’s Hospital is a university-affiliated tertiary facility that provides post-transplant care to approximately 1600 adult renal transplant recipients. As shown in Fig. 1, we identified a cohort of all de novo renal transplant recipients who received a living or deceased donor allograft between the period of January 1, 2005 and December 31, 2010 and had a minimum of 6-month post-transplant follow-up. Data was obtained from an electronic renal transplant database. Data was collected retrospectively for demographic and baseline attributes and to identify recipients who were prescribed an ESA. ESA exposure measurement included time of the first dose of ESA post-transplant within < 3, 3–6, and >6 months from transplant, as well as total duration of treatment post-transplant <3, 3–6, and >6 months. NODAT was identified through routine post-transplant blood glucose screening and defined by a fasting blood glucose (FBG) ≥7.0 mmol/L and/or random blood glucose (RBG) ≥11.1 mmol/L on two separate occasions in the absence of acute illness as per the 2008 Canadian Diabetes Association diagnostic criteria [22]. Of note, there were no female participants who became pregnant and developed gestational diabetes. Univariate comparisons for transplant demographic/baseline characteristics and outcome variables between ESA exposure or no ESA exposure and those who developed NODAT or did not were done by chi-square analysis or Fisher’s exact test as appropriate. Multivariate logistic regression models were then constructed using ESA, age, donor source, random blood glucose (RBG) at discharge, acute rejection (AR), and body mass index (BMI) as independent variables and univariate factors with *p* < 0.20 as other independent variables, and NODAT as the dependent variable. Immunosuppressive profile was based on the initial hospital discharge summary. Patients with any prior history of diabetes or glucose intolerance or had multi-organ transplant including a second renal transplant or more were excluded. Institutional Review Board approval was obtained (Research ethics board #10-313). SAS was the statistical software used for the analysis.

## Results

Six hundred fifteen recipients met initial criteria of which 164 were excluded. Of those excluded, 147 had a previous history of diabetes mellitus prior to transplant, two had impaired glucose tolerance, one patient had follow-up less than 6 months post-transplant, four patients had a second renal transplant, one patient had a failed renal transplant, and nine patients had NODAT prior to ESA initiation. In the remaining 451 recipients who were transplanted between January 1, 2005 and December 31, 2010, 132 were exposed to an ESA at any time post-transplant and 319 were not exposed. Of those exposed to an ESA, there was a significant reduction in the development of NODAT with four developing NODAT compared to 128 who did not develop NODAT (*p* < 0.0001). Thus, there was only a 3 % (4/132) chance of developing NODAT on an ESA compared to 15 % (48/319) chance not on an ESA. Of the 132 patients who received an ESA, none of the 29 patients who received ESA for up to 3 months duration at any time post transplant developed NODAT (*p* = 0.025). While 1 of the 44 patients who received an ESA for up to 6 months duration at anytime post transplant developed NODAT (*p* = 0.0006). Three of the 56 patients who received an ESA >6 months duration at any time post transplant developed NODAT; however, this did not reach statistical significance (*p* = 0.065). Early exposure did not appear protective when ESA was given within 3 months post transplant (*p* = 0.23).

There are two ESAs currently available in Canada, namely epoetin alfa (Eprex) and darbepoetin (Aranesp). Of the 132 recipients exposed to an ESA, 75 % received darbepoetin (Aranesp) and 25 % received epoetin alfa (Eprex).

### ESA vs. no ESA

Those on an ESA had significantly higher serum creatinine at discharge (*p* = 0.0002), 3 months (*p* = 0.005) and 6 months (*p* = 0.001), lower eGFR, and lower hemoglobin compared to those not on an ESA. Hemoglobin at discharge on an ESA was 93.8 ± 14.8 (67–131) vs. 100.8 ± 15 (69–149) not on an ESA, *p* < 0.0001. Hemoglobin at 6 months post-transplant was 123.4 ± 17.4 (63–163) on an ESA vs. 131.3 ± 17.5 (65–183) not on an ESA, *p* < 0.0001. Those on an ESA were more likely to have been recipients of deceased donor (*p* = 0.005) and on azathioprine (*p* = 0.01). Those on MMF (*p* = 0.004), cyclosporine (*p* = 0.02), or a statin (*p* = 0.02), and male gender (*p* = 0.002) tended not to be on ESA. Prednisone dose at discharge was lower in those on an ESA (27.2 mg ± 14.4) compared to those not on an ESA (30.8 mg ± 17) (*p* = 0.035). However, prednisone dose was not significant at 3 months (8 mg ± 10.2 on ESA and 6.7 mg ± 4.4 not on an ESA) and 6 months (5.5 mg ± 2.7 on ESA and 5.6 mg ± 3.1 not on an ESA) post transplant (*p* = 0.18 and *p* = 0.68). There was no significant difference in fasting blood glucose (FBG) at discharge (5.9 mmol/L ± 1.2 and 5.6 mmol/L ± 1.3, *p* = 0.23), 3 months (5.4 mmol/L ± 0.9 and 5.4 mmol/L ± 0.6, *p* = 0.82) and 6 months (5.5 mmol/L ± 0.8 and 5.4 mmol/L ± 0.6, *p* = 0.80) between the two groups.

### NODAT vs. no NODAT

Baseline demographics at the time of transplant (*N* = 451) of older age (*p* = 0.0001), hypertension as a cause of end-stage renal disease (*p* = 0.003), prednisone (*p* = 0.02), and ASA (*p* < 0.0001) were statistically significant between the NODAT and no NODAT groups, where the risk of NODAT was higher (see Table 1—not all data is shown). In the 399 patients who had no NODAT, immunosuppression consisted of tacrolimus (311), tacrolimus extended release (73), cyclosporine (55), MMF (299), Myfortic (53), prednisone (345), azathioprine [16], and Rapamune [23]. Prednisone dose was not significant at discharge (31.6 mg ± 12 with NODAT and 29.4 mg ±16.8 with NoNODAT, *p* = 0.26), at 3 months (7.3 mg ± 5 with NODAT and 7.1 mg ± 7.1 with no NODAT, *p* = 0.88) and 6 months (6.3 mg ± 4.4 with NODAT and 5.5 mg ± 2.7 with no NODAT, *p* = 0.10) post transplant between the two groups. Two patients were on a prednisone avoidance immunosuppressive regimen, neither of which was on an ESA. One patient was not given prednisone due to the risk of GI bleed and the other was due to the strong family history of type 2 diabetes. Despite not being on prednisone, the patients still got NODAT.

**Table 1:** Univariate analysis. Baseline characteristics/demographics

Baseline	NODAT	No NODAT	*p* value	ESA	No ESA	*p* value
	*N* = 52	*N* = 399		*N* = 132	*N* = 319	
ESA exposure (%)	4 (3)	128 (97)		–	–	<0.0001
NODAT Yes (%)	–	–		4 (8)	48 (92)	–
Age at transplant (years)	58.2 ± 11.6	50.7 ± 13.4	0.0001	51.8 ± 13.8	51.4 ± 13.2	0.51
Male (%)	33 (63)	248 (62)	0.89	68 (51)	213 (67)	0.002
Donor source deceased (%)	33 (63)	187 (47)	0.005	78/ (60)	142 (45)	0.005
Body mass index (BMI)	26.9 ± 6.5	26.2 ± 5	0.43	25.8 ± 4.9	26.5 ± 5.3	0.21
Hepatitis C+ yes (%)	0 (0)	4 (1)	0.76	1 (0.7)	3(0.9)	0.63
Acute rejection (AR) yes (%)	2 (4)	26 (6)	0.35	11 (8)	17(5)	0.22
Cause end-stage renal disease:						
Hypertension	11	33	0.003	11	33	0.51
Glomerulonephritis	25	196	0.88	70	151	0.27
Polycystic kidney disease	6	55	0.65	16	45	0.57
Interstitial nephritis	2	46	0.06	14	34	0.98
Obstructive uropathy	1	6	0.57	1	6	0.34
Other	7	63	0.66	20	50	0.88
Blood glucose at start of ESA (*N* = 13/*N* = 129)	7.3 ± 2	6.2 ± 1.4	0.04	–	–	–
Blood glucose at the end of ESA (*N* = 13/*N* = 129)	5.3 ± 1.5	5.7 ± 1.2	0.08	–	–	–
Tacrolimus IR yes (%)	40 (77)	311 (78)	0.86	104 (79)	247 (77)	0.75
Cyclosporine yes (%)	9 (17)	55 (14)	0.49	11 (8)	53 (16)	0.02
Tacrolimus ER (Advagraf) yes (%)	8 (15)	74 (18.5)	0.67	20 (15)	61 (19)	0.31
Prednisone yes (%)	50 (96)	345 (86)	0.02	121 (92)	274 (86)	0.09
Creatinine (SCr) at initial hospital discharge (umol/L)	162.8 ± 110.2	180.4 ± 147.7	0.32	225.1 ± 188.3	157.5 ± 113	0.0002
SCr at 3 months (umol/L)	114.3 ± 35.2	127.2 ± 43.6	0.05	136.4 ± 56.5	121.1 ± 34.4	0.005
SCr at 6 months (umol/L)	119.3 ± 36.7	128.9 ± 49.8	0.10	142.9 ± 70.3	121.1 ± 32.6	0.001
Hemoglobin (Hb) at discharge	98 ± 17.8	98.7 ± 14.9	0.76	93.8 ± 14.8	100.8 ± 15	<.0001
Hb at 3 months	120.8 ± 16.8	123 ± 17.2	0.41	117.7 ± 16.2	125 ± 16.9	<.0001
Hb at 6 months	131.6 ± 14.4	128.5 ± 18.2	0.25	123.4 ± 17.3	131.3 ± 17.5	<.0001
Random blood glucose (RBG) at discharge	7.1 ± 2.1	5.8 ± 1.7	<.0001	6 ± 1.8	6 ± 1.8	0.93
RBG at 3 months	7.7 ± 2.3	5.9 ± 1.3	<.0001	6.2 ± 1.6	6.1 ± 1.7	0.65
RBG at 6 months	6.9 ± 1.6	5.6 ± 0.9	<.0001	5.5 ± 1	5.8 ± 1.2	0.01

*ESAs* erythropoietin-stimulating agents, *NODAT* new-onset diabetes after renal transplant, *RBG* random blood glucose, *BMI* body mass index, *Hb* hemoglobin, *SCr* serum creatinine

FBG at discharge (5.9 mmol/L ± 1.8 and 5.6 mmol/L ± 1.2, *p* = 0.0.519), 3 months (5.9 mmol/L ± 0.4 and 5.4 mmol/L ± 0.7, *p* = 0.25) or 6 months (5.8 mmol/L ± 1 and 5.4 mmol/L ± 0.7, *p* = 0.18) post transplant was not significant between the two groups. Demographic and other characteristics between those with NODAT and those with no NODAT are provided in Table 1.

We examined NODAT rates 6 months after transplant and compared those exposed to an ESA vs. those who were not exposed to an ESA by chi-square analysis. Of the 132 patients who were exposed to an ESA, two developed NODAT. Of the 319 patients who were not exposed to an ESA, 15 developed NODAT. However, this was not statistically significant (*p* = 0.08). Therefore, early exposure to ESA does not seem to protect against NODAT at 6 months post transplant.

As shown in Table 2, the final multivariate logistic regression model, being on an ESA at any time post-transplant reduced the risk of developing NODAT; OR = 0.08, 95 % CI (0.018–0.352), *p* = 0.0008. Older age; OR = 1.41, 95 % CI (1.036–1.933), *p* < 0.02, higher random blood sugar at discharge; OR = 1.30, 95 % CI (1.077–1.57), *p <* 0.006 and deceased donor; OR = 2.18 CI (1.009–4.729), *p* = 0.04 were associated with an increased risk of NODAT. The overall NODAT rate was 11.5 % (52/451). Although there was no difference in mean time-to-NODAT on an ESA, 770 days (SD 863) vs. 249 days (SD 431) not on an ESA, *p* = 0.31, there was a trend in delaying the onset of diabetes in those on an ESA. Mean time from transplant to the first dose of ESA was 153 days. Mean duration of follow-up was 1141.9 days (SD = 590.6). Seventeen patients were on an ESA pre-transplant, none of whom developed NODAT. However, two were restarted on an ESA post transplant, for less than 3 months and the other greater 6 months.

**Table 2:** Final multivariate logistic regression model. ESA exposure at any time post transplant

Parameter	Odds ratio estimate	95 % Wald confidence limits	*p* value
ESA	0.08	0.018–0.352	0.0008
Donor source (deceased vs. live)	2.18	1.009–4.729	0.04
Age (per 10 years)	1.41	1.036–1.933	0.02
Random blood glucose (RBG) at discharge	1.30	1.077–1.57	0.006
Body mass index (BMI)	1.04	0.974–1.124	0.21
Acute rejection (AR)	1.21	0.129–11.423	0.86

*ESA* erthropoietin stimulating agents, *RBG* random blood glucose, *BMI* body mass index, *AR* acute rejection

There were 29 patients from the original cohort that were no longer followed in our clinic by the end of the study period. Twelve patients were deceased, 14 returned to dialysis due to graft failure, and 3 were discharged from our clinic/transferred to another facility for their post-transplant care. Of the 29 patients who were no longer followed, 22 were exposed to an ESA, 19 of which did not have NODAT.

## Discussion

Exposure to an ESA at any time post transplant, particularly if given for up to 6 months duration, was associated with a reduced risk of developing NODAT compared to those who were not exposed. The mechanism of this effect is unclear. It is also unclear whether exposure to ESA for greater than 6-month duration impacts the risk of NODAT. In our study and in other analyses, the highest risk of NODAT was within the first 12 months post-transplant.

ESAs have decreased the need for blood transfusions and improved the quality of life in patients with CKD and anemia compared to no treatment; however, the risks and benefits of ESA therapy as well as cost effectiveness is unclear in renal transplant recipients due to the limited evidence available [24–26]. The Correction of Anemia and Progression of Renal Insufficiency in Transplanted (CAPRIT) patients study, a European, multicenter, randomized trial of renal transplant recipients assessed the effect of normalization of anemia (130–150 g/L) compared to partial correction (105–115 g/L) with ESA on the progression of graft dysfunction. The CAPRIT authors found after 2 years of follow-up that, normalization of hemoglobin was associated with improved quality of life, and less progression of chronic allograft nephropathy without an increase risk of cardiovascular events [23]. However, in a retrospective study, an increase in mortality was seen in renal transplant recipients who had hemoglobin levels lower or higher than 125 g/L on an ESA (U-shaped curve) compared to those not on an ESA, where the relationship between hemoglobin and mortality was linear [26]. Studies targeting normal or higher hemoglobin levels in patients with CKD showed an increased risk of adverse outcomes including cerebrovascular events, vascular access thrombosis and hypertension, and an increase in cost compared to a lower hemoglobin target [25, 27, 28]. It has also been postulated that higher ESA doses may increase the risk of mortality; however, this has yet to be confirmed [29]. Therefore, weighing potential benefits of ESA in reducing the risk of NODAT must be weighed against the potential adverse effects particularly when the hemoglobin is near normal and at higher doses of ESA.

Limitations of this study include its retrospective nature, single center, and homogenous population; thus, generalizability of the results must be approached with caution. Although several confounders are included in the multivariate regression model, we did not include other potentially important confounders such as gender, ethnicity, hepatitis C, and steroid use in the analysis and therefore residual confounding may exist. In addition, immunosuppression may have been altered by the prescriber in response to blood sugar levels to minimize risk of NODAT, thus leading to selection bias.

## Conclusions

The incidence of NODAT and its effects in renal transplant recipients is significant, including greater risk of graft loss and reduced patient survival. In our study, we found that older age, higher RBG at discharge, and deceased donor were associated with an increased risk of NODAT; however, even accounting for these factors, ESA exposure remained protective. Reducing the risk of NODAT may improve the overall health and quality of life of patients and their renal transplant. Being on an ESA may have a role in preventing NODAT, particularly if given for up to 6 months duration post-transplant, taking into account the overall risks and benefits of ESA therapy in the renal transplant population, although this remains hypothesis generating.

## References

[1] Miles AMV, Sumrani M, Horowitz R, Home P, Maursky V, Markell M (1998). Diabetes mellitus after renal transplantation: as deleterious as non-transplant-associated diabetes?. Transplantation.

[2] Jindal RM, Hjelmesaeth J (2000). Impact and management of post-transplant diabetes mellitus. Transplantation.

[3] Davidson J, Wilkinson A, Dantal D, Dotta F, Haller H, Hernandez D (2003). New-onset diabetes after transplantation: 2003 international consensus guidelines. Transplantation.

[4] Valderhaug TG, Jenssen T, Hartmann A, Midtvedt K, Holdaas H, Reisaeter AV (2009). Fasting plasma glucose and glycosylated hemoglobin in the screening for diabetes mellitus after renal transplantation. J Transplant.

[5] David-Neto E, Lemos FC, Fadel LM, Agena F, Sato MY, Coccuza C (2007). The dynamics of glucose metabolism under calcineurin inhibitors in the first year after renal transplantation in nonobese patients. Transplantation.

[6] Porrini E, Moreno JM, Osuna A, Benitez R, Lampreabe I, Diaz JM (2008). Prediabetes in patients receiving tacrolimus in the first year after kidney transplantation: a prospective and multicenter study. Transplantation.

[7] Crutchlow MF, Bloom RD (2007). Transplant-associated hyperglycemia: a new look at an old problem. Clin J Am Soc Nephrol.

[8] Ghisdal L, VanLaecke S, Abramowicz M, Vanholder R, Abramowicz D (2012). New-onset diabetes after renal transplantation. Risk assessment and management. Diabetes Care.

[9] Kidney Disease: Improving Global Outcomes (KDIGO) Anemia Work Group (2012). KDIGO Clinical Practice Guideline for anemia in chronic kidney disease. Kidney Int.

[10] National Kidney Foundation (2006). KDOQI Clinical Practice Guidelines and Clinical Practice Recommendations for anemia in chronic kidney disease. Am J Kidney Dis.

[11] Coyne D, Brennan D (2013). Anemia and the renal transplant recipient.

[12] Banag A, Yousif M, Elmusharaf K (2011). Risk factors of post renal transplant anemia among Sudanese patients, a study in three renal transplant centers. BMC Nephrol.

[13] Meng R, Zhu D, Bi Y, Yang D, Wang Y (2013). Erythropoietin inhibits gluconeogenesis and inflammation in the liver and improves glucose intolerance in high-fat diet-fed mice. PLoS One.

[14] Choi D, Schroer S, Lu S, Wang L, Wu X, Liu Y (2010). Erythropoietin protects against diabetes through direct effects on pancreatic β cells. J Exp Med.

[15] Fenjves ES, Ochoa MS, Cabrera O, Mendez AJ, Kenyon NS, Inverardi L (2003). Human, nonhuman primate, and rat pancreatic islets express erythropoietin receptors. Transplantation.

[16] Scully MS, Ort T, James I, Bugelski PJ,.Makropoulos DA, Deutsch HA et al., A novel EPO receptor agonist improves glucose tolerance via glucose uptake in skeletal muscle in a mouse model of diabetes. Exp Diabetes Res. 2011; doi: 10.1155/2011/910159 DOI:10.1155%2F2011%2F910159 .10.1155/2011/910159 PMC3132469 21754921

[17] Allegra V, Mengozzi G, Martimbianco L, Vasile A (1996). Early and late effects of erythropoietin on glucose metabolism in maintenance hemodialysis patients. Am J Nephrol.

[18] Pan Y, Shu J, Ghu H, Zhou D, Liu XL, Qiao QY (2013). Erythropoietin improves insulin resistance via the regulation of its receptor-mediated signaling pathways in 3T3L1 adipocytes. Mol Cell Endocrinol.

[19] Youssoufian H, Longmore G, Neuman D, Yoshimura A, Lodish HF (1993). Structure, function and activation of the erythropoietin receptor. Blood.

[20] Shuai H, Zhang J, Yu Y, Zhang M (2008). Expression of EPO receptor in pancreatic cells and its effect on cell apoptosis. J Huazhong Univ Sci Technol.

[21] Shah R, Chang Y, Woo M, Connelly P, Hanley A, Sermer M (2015). Erythropoietin and glucose homeostasis in women at varying degrees of future diabetic risk. J Diabetes Complications.

[22] Canadian Diabetes Association Clinical Practice Guidelines Expert Committee (2008). Canadian Diabetes Association 2008 clinical practice guidelines for the prevention and management of diabetes in Canada. Can J Diabetes.

[23] Choukroun G, Kamar N, Dussol B, Etienne I, Cassuto-Viguier E (2012). Correction of post kidney transplant anemia reduces progression of allograft nephropathy. J Am Soc Nephrol.

[24] Clement F, Klarenbach S, Tonelli M, Wiebe N, Hemmelgarn B (2010). An economic evaluation of erythropoiesis-stimulating agents in CKD. Am J Kidney Dis.

[25] Ferguson T, Xu Y, Gunasekara R, Lerner B, Macdonald K (2015). The cost effectiveness of erythropoietin-stimulating agents for treating anemia in patients on dialysis: a systematic review. Am J Nephrol.

[26] Yabu J, Winkelmayer W (2011). Post transplantation anemia: mechanisms and management. Clin J Am Soc Nephrol.

[27] Palmer S, Saglimbene V, Mavridis D, Salanti G, Craig J (2014). Erythropoiesis-stimulating agents for anemia in adults with chronic kidney disease: a network meta-analysis. Cochrane Database Syst Rev.

[28] Vinhas J, Barreto C, Assunção J, Parreira L, Vaz A (2012). Treatment of anemia with erythropoiesis-stimulating agents in patients with chronic kidney disease does not lower mortality and may increase cardiovascular risk: a meta-analysis. Nephron Clin Pract.

[29] Szczech LA, Barnhart HX, Inrig JK, Reddan DN, Sapp S (2008). Secondary analysis of the CHOIR trial epoetin-alpha dose and achieved hemoglobin outcomes. Kidney Int.

